# The efficacy and safety of lenvatinib plus transarterial chemoembolization in combination with PD-1 antibody in treatment of unresectable recurrent hepatocellular carcinoma: a case series report

**DOI:** 10.3389/fonc.2023.1096955

**Published:** 2023-05-16

**Authors:** Chunyang Mu, Junyi Shen, Xinrui Zhu, Wei Peng, Xiaoyun Zhang, Tianfu Wen

**Affiliations:** ^1^Liver Surgery/Liver Transplantation Center, West China Hospital, Sichuan University, Chengdu, Sichuan, China; ^2^Department of Hepatobiliary and Pancreatic Surgery, The Third Affiliated Hospital of Suzhou University, Changzhou, Jiangsu, China

**Keywords:** recurrent hepatocellular carcinoma, lenvatinib, trans-arterial chemoembolization, programmed death-1 antibody, combination therapy

## Abstract

**Purpose:**

To explore the safety and efficacy of lenvatinib in combination with trans-arterial chemoembolization (TACE) and programmed death receptor 1 (PD-1) antibody in the treatment of unresectable recurrent hepatocellular carcinoma (urHCC).

**Patients and methods:**

The clinical data of 16 patients with unresectable recurrent hepatocellular carcinoma admitted to the Department of Liver Surgery and Liver Transplantation Center, West China Hospital, Sichuan University, and received the conversion therapy of lenvatinib + TACE + PD-1 antibody between January 2019 and January 2022 were retrospectively analyzed.

**Results:**

There were 25% (4/16) patients suffering from grade 3 adverse events and no patients suffering from grade 4 or higher adverse events. After 4 months of treatment of 16 patients, according to the modified Response Evaluation Criteria in Solid Tumors (mRECIST), two, five, three, and six cases were in complete response (CR), partial response (PR), stable disease (SD), and progressive disease (PD), respectively, and the objective response rate (ORR) was 43.8% (7/16). The 1-year overall survival (OS) rate and 1-year progression-free survival (PFS) rate were 86.2% and 46.9%, respectively. In our subgroup analysis, the ORR of patients with multiple lesions reached up to 60%, which was higher than that of patients with single lesions.

**Conclusions:**

Lenvatinib in combination with TACE and PD-1 antibody is safe and effective in the treatment of unresectable recurrent hepatocellular carcinoma.

## Introduction

Hepatocellular carcinoma (HCC) is the fourth leading cause of death worldwide. Surgical resection is the main radical therapy of early-stage HCC (1). However, approximately 70% of patients eventually suffer from postoperative recurrence within 5 years (2). For unresectable recurrent HCC patients, there were no universal and effective treatment options due to complicated factors including the limited reserve of liver function, severe postoperative complications, and multifocal recurrent tumors or extrahepatic metastasis (3). Hence, it was of great importance to explore new treatment modalities for patients suffering from unresectable recurrent HCC (urHCC).

The trans-arterial chemoembolization (TACE) is the standard treatment for advanced-stage HCC patients and significantly improves prognosis *via* targeted chemotherapy and ischemic necrosis way (4). In clinics, TACE has also been widely used for urHCC patients (5, 6). Additionally, multi-kinase inhibitor lenvatinib is the first-line approach for patients with advanced HCC because of its effective role in remodeling tumor blood vessels and inhibiting tumor growth (7). In recent studies, the combination of TACE and multi-kinase inhibitors has been shown to be superior to monotherapy (8). Recently, immune checkpoint inhibitors (ICIs) are becoming a promising anti-tumor approach for HCC patients. The combination of multi-kinase inhibitor with ICI could achieve an objective response rate (ORR) of approximately 30%, higher than that of monotherapy (9, 10). Particularly, our recent study reported that a triplet regimen, namely, combinations of TACE, lenvatinib, and PD1, provided satisfactory efficacy and safety in the treatment of advanced-stage HCC. Some patients could even achieve eligibility for surgical resection (11). However, the role of a triplet regimen in recurrent HCC remained unclear.

Here, this retrospective study investigated the safety and efficacy of TACE plus lenvatinib in combination with anti-PD-1 in the treatment of unresectable recurrent HCC.

## Material and methods

### Study design and patient selection

We reviewed the clinicopathological features of 16 patients with unresectable recurrent HCC who accepted treatment with TACE + lenvatinib and anti-PD-1 antibody between January 2019 and January 2022 at West China Hospital of Sichuan University. The study was conducted in accordance with the Declaration of Helsinki (as revised in 2013) and was approved by the Ethics Committee of West China Hospital, Sichuan University. Written informed consent for the patients’ data to be used for research was obtained from all patients prior to treatment. All the patients included in this study met the following inclusion criteria: 1) age between 18 and 75 who underwent R0 hepatectomy with pathologically proven HCC; 2) the relapsed tumor was considered as unresectable after multidisciplinary discussion (the criteria of unresectable HCC was defined as any of the following: the residual liver volume after re-operation is insufficient; multifocal recurrent tumors; 3) Eastern Cooperative Oncology Group performance status (ECOG PS) score of 0 or 1; 4) Child–Pugh class A or B liver function; 5) at least one measurable lesion ≥1 cm in the liver based on the modified Response Evaluation Criteria in Solid Tumors (mRECIST). The exclusion criteria were 1) incomplete clinical information, 2) loss to follow-up, and 3) ECOG > 1.

### Therapeutic schedule

Lenvatinib (8 mg if bodyweight < 60 kg; 12 mg if bodyweight ≥ 60 kg) was taken orally once a day prior to TACE. Two to three weeks after TACE, an anti-PD-1 antibody was injected intravenously at 200 mg every 2 or 3 weeks. Computed tomography (CT) or magnetic resonance imaging (MRI) evaluation of epigastric enhancement was performed 1 month after each session, and the doctor determined whether and when the next TACE session would be performed.

### Assessment of clinical outcome and follow-up

All the patients in this cohort were followed up until 31 May 2022. The patients underwent laboratory tests and contrast-enhanced CT of the upper abdominal every 6–8 weeks to assess treatment adverse effects and efficacy. The overall survival (OS), progression-free survival (PFS), disease control rate (DCR), and adverse events were assessed. The OS was calculated from the first day after the combination therapy to all-cause death. PFS was calculated from the first day after the treatment to progression of disease or death during the treatment. The tumor response was evaluated according to mRECIST, whereas adverse events were defined according to the National Cancer Institute—Common Terminology Criteria for Adverse Events (NCI-CTCAE) version 5.0. The DCR was defined as the percentage of patients who achieved complete response, partial response, and stable disease after the treatment.

### Statistical analysis

R-4.0.2 statistical software was used for all statistical analyses and data visualizations. Quantitative data were expressed as the means ± standard deviation or median (first–third quartile), whereas categorical variables were expressed as a proportion. The Kaplan–Meier curves were used to illustrate the PFS and OS.

## Results

### The characteristics of patients

A total of 16 patients with complete follow-up information were enrolled in this study. The baseline information of the 16 patients is listed in **Table 1**. Fourteen out of the 16 patients (87.5%) were male with a mean age of 55.8 ± 10.6 years. The mean weight of these patients was 62.3 ± 9.3 kg, and 13 patients had previous hepatitis B virus (HBV) infection history. The mean tumor size of the relapsed HCC was 4.5 ± 3.6 cm. Ten out of the 16 patients (62.5%) presented multiple tumors. Two patients had portal vein tumor thrombus. The median level of alpha-fetoprotein (AFP) and PIVKA-II was 8.22 ng/ml and 82 mAU/ml, respectively. Furthermore, all the patients had an adequate hematopoietic reserve and nearly normal liver function. Pathological features of the primary tumor are listed in **Table 2**.

**Table 1:** The baseline characteristics of the 16 enrolled patients.

Characteristic		Value
Age (x ± s, year)		55.8 ± 10.6
Sex		
Male		14 (87.5%)
Female		2 (12.5%)
Weight (x ± s, kg)		62.3 ± 9.3
Chronic HBV infection (%)		
Yes		13 (81.3%)
No		3 (18.7%)
ECOG PS score (%)		
0		13 (81.3%)
1		3 (18.7%)
Number of tumors (%)		
1		6 (37.5%)
≥2		10 (62.5%)
Tumor size (%)		
<5 cm		12 (75%)
≥5 cm		4 (25%)
Portal vein invasion (%)		
Yes		2 (12.5%)
No		14 (87.5%)
AFP [M (*P25*, *P75*), ng/ml]		8.22 (4.85, 26.7)
PIVKA-II [M (*P25*, *P75*), mAU/ml]		82 (27.3, 255.3)
TB (x ± s, μmol/L)		13.6 ± 6.7
ALB (x ± s, g/L)		38.8 ± 14.1
Child–Pugh grade (%)		
A		16 (100%)
B/C		0 (0%)
Hb (x ± s, g/L)		139.4 ± 30.1
WBC (x ± s, ×10^9^/L)		5.0 ± 1.4
PLT (x ± s, ×10^9^/L)		116.8 ± 33.1
Time to recurrence (x ± s, month)		15.2 ± 14.4

AFP, α-fetoprotein; PIVKA-II, protein induced by vitamin K absence-II; TB, total bilirubin; ALB, albumin; Hb, hemoglobin; WBC, white blood cell; PLT, platelet; HBV, hepatitis B virus; ECOG PS, Eastern Cooperative Oncology Group performance status.

**Table 2:** The pathological features of the corresponding primary tumors.

Characteristic				Value	
Tumor size (x ± s, cm)				5.3 ± 2.6	
Number of tumors (%)					
1				10 (62.5%)	
≥2				6 (37.5%)	
Intact tumor capsule (%)					
Yes				11 (68.8%)	
No				5 (31.2%)	
Clear tumor boundary (%)					
Yes				12 (75%)	
No				4 (25%)	
MVI (%)					
M0				8 (50%)	
M1				5 (31.3%)	
M2				3 (18.7%)	
Grade (%)					
G2				9 (56.3%)	
G2–3				5 (31.3%)	
G3				2 (12.5%)	

MVI, microvascular invasion.

### Safety of the triplet combination therapy

All 16 patients experienced some degree of adverse events as listed in **Table 3**, and the most common adverse reaction was hypertension (50%), abdominal pain (50%), thrombocytopenia (50%), diarrhea (37.5%), fatigue (37.5%), etc. No grade 4 or above adverse events were observed in them, and four out of 16 patients (25%) suffered grade 3 adverse events. Specifically, the dosage of lenvatinib was reduced in two patients (cases 4 and 8) for thrombocytopenia, and avatrombopag was administered for them to recover their platelets; the treatment of one patient (case 2) was changed to apatinib because of intolerable hypertension; one patient (case 9) had a dose reduction of lenvatinib because of severe hand-foot syndrome. The adverse events in other patients were managed symptomatically without dosage reduction, discontinuation, or change of medication.

**Table 3:** The adverse events (AEs) during the treatment.

AEs	Grade 1–2 (%)	Grade 3–4 (%)	Total (%)
Hand-foot skin reaction	2 (12.5)	2 (12.5)	4 (25)
Diarrhea	6 (37.5)	0 (0)	6 (37.5)
Hypertension	7 (43.8)	1 (6.3)	8 (50.0)
Loss of appetite	4 (25.0)	0 (0)	4 (25.0)
Weight loss	4 (25.0)	0 (0)	4 (25.0)
Fatigue	6 (37.5)	0 (0)	6 (37.5)
Proteinuria	3 (18.8)	0 (0)	3 (18.8)
Abdominal pain	7 (43.8)	1 (6.3)	8 (50.0)
Platelet decrease	6 (37.5)	2 (12.5)	8 (50.0)
ALT increase	2 (12.5)	1 (6.3)	3 (18.8)
AST increase	3 (18.8)	1 (6.3)	4 (25.0)
Hyperbilirubinemia	2 (12.5)	0 (0)	2 (12.5)
Fever	4 (25.0)	0 (0)	4 (25.0)
Hypothyroidism	1 (6.3)	0 (0)	1 (6.3)

ALT, alanine aminotransferases; AST, aspartate aminotransferases.

### Efficacy of the triplet combination therapy

**Figure 1:**
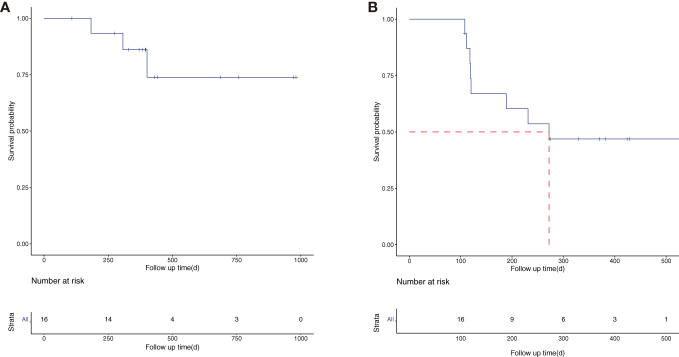
The overall survival (OS) **(A)** and progression-free survival (PFS) **(B)** curve of the 16 enrolled patients.

The total follow-up time for the 16 patients was 120–1,093 days, with a median follow-up time of 397 days. The median OS time was not reached, and the 1-year OS rate was 86.2% [95% CI (70.0%, 92.4%)] (**Figure 1A**). The median PFS was 272 days, and the 1-year PFS rate was 46.9% [95% CI (27.3%, 80.3%)] (**Figure 1B**). The subgroup analysis was performed according to the number of lesions. The patients were divided into two groups: multiple lesions (10 cases) and single lesions (six cases). The 1-year OS rate and 1-year PFS rate were 88.9% and 60.0% respectively in the multiple lesions group; the corresponding 1-year OS rate and 1-year PFS rate of the single-lesion group were 75.0% and 16.7%, respectively.

**Figure 2:**
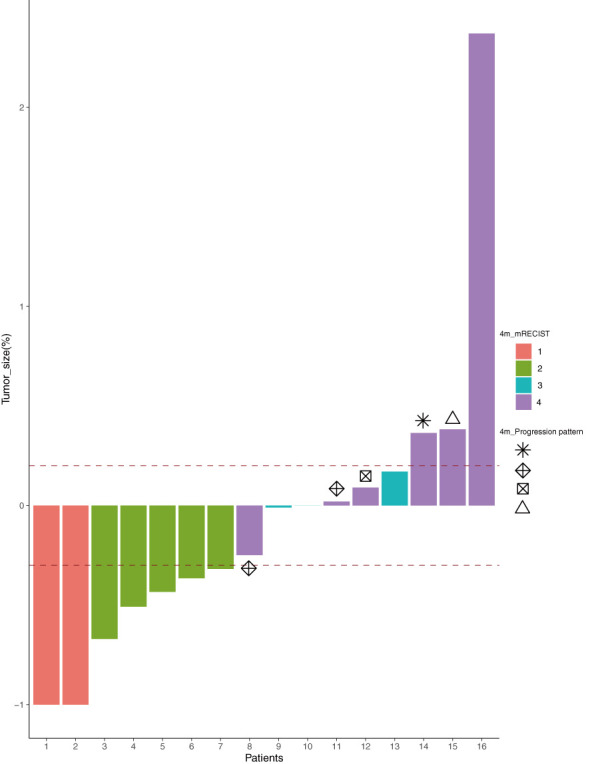
The tumor response according to the mRECIST (1, CR; 2, PR; 3, SD; 4, PD) after 4 months of treatment and the progression pattern (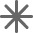, target; 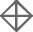, new lesions; 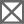, non-target; 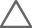, else) of the six PD patients. mRECIST, modified Response Evaluation Criteria in Solid Tumors; CR, complete response; PR, partial response; SD, stable disease; PD, progressive disease.

After 4 months of treatment with lenvatinib + TACE + PD-1 mAb, the tumor response was evaluated according to the mRECIST (**Figure 2**). Of the 16 patients, two had complete response (CR), five had partial response (PR), three had stable disease (SD), and six had disease progression (PD), resulting in an objective response rate of 43.8%. In the subgroup analysis, the patients in the multiple lesions group had a higher ORR when compared with the single-lesion group [60.0% (6/10) *vs.* 16.7% (1/6), p = 0.145].

### Progression pattern and post-progression treatment schedule

We also reviewed the pattern of progression and post-progression treatment for all patients. Six patients had disease progression after four sessions of treatment, and their progression patterns were as follows (as shown in **Figure 2**): two with progression of target lesions only, one with progression of non-target lesions only, two with progression of new lesions only, and one with progression of target + non-target + new lesions simultaneously. A total of nine patients had worsening disease at the final follow-up. Their treatment schedules after disease progression were decided by multidisciplinary discussion, and the majority of patients (12/15) accepted the multidisciplinary team (MDT) recommended schedule, except for cases 7, 16, and 19.

### Presentation of three patients with better outcomes

**Figure 3:**
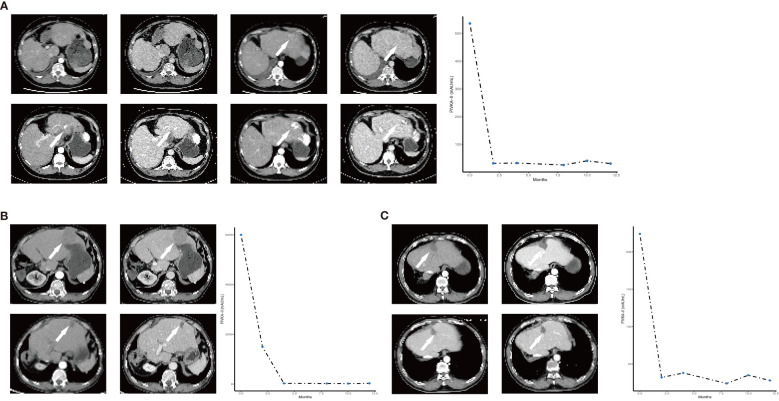
The representative CT images pre- and post-treatment and the change of PIVKA-II during the treatment schedule of the three patients with better outcome (**A–C**: case 1-3). The arrow indicated that the lesions change with the treatment.

Case 1 was a 40-year-old male patient suffering from unresectable recurrent HCC with multiple lesions. After 6.5 months of therapy with lenvatinib (12 mg), TACE (two times), and camrelizumab (200 mg), the tumor diameter decreased from 10.36 to 5.87 cm, and the level of PIVKA-II decreased from 536 to 26 mAU/ml (**Figure 3A**). The patient, who achieved partial response according to the mRECIST, eventually underwent hepatectomy, and he is still living well and being followed up regularly.

Case 2 was a 73-year-old male patient with two lesions in his residual liver. After 4 months of therapy with lenvatinib (8 mg), TACE (one time), and camrelizumab (200 mg), the tumor nearly disappeared with a sharp decline of PIVKA-II. He achieved complete response according to mRECIST (**Figure 3B**).

Case 3 was a 60-year-old male patient suffering from urHCC with a single lesion in the residual liver. After 4 months of therapy with lenvatinib (8 mg), TACE (one time), and camrelizumab (200 mg), the tumor disappeared, and the PIVKA-II decreased from 224 to 38 mAU/ml. The patient achieved complete response according to mRECIST (**Figure 3C**).

## Discussion

Post-surgical recurrence of hepatocellular carcinoma is an urgent issue to be solved in clinical practice, and various therapies, including rehepatectomy, salvage liver transplantation, and radiofrequency ablation, have been proven to be able to achieve radical treatment in some patients with low tumor burden (3). However, for the majority of patients with an advanced-stage unresectable tumor, there is no consensus on the standard treatment. These patients had to choose palliative approaches with limited efficacy, such as TACE, sorafenib, and lenvatinib, to prolong their life (3). Inspiringly, the combination of local regional therapy, special TACE, and systemic approaches is being widely tested in the management of advanced HCC. It has been demonstrated that lenvatinib in combination with TACE could improve the clinical outcome of advanced HCC patients (8). Additionally, the data from our center also confirm the superiority of lenvatinib plus TACE in combination with PD-1 mAb (LEN-TAP) in the treatment of patients with advanced HCC (10). Some preclinical studies have demonstrated the synergies between antiangiogenic agents, ICIs, and local regional therapies in mouse HCC models (12–15). Additionally, an interesting study exploring the ecosystem of recurrent hepatocellular carcinoma reveals a unique immune cell status in its tumor microenvironment (16). Therefore, we speculate that the LEN-TAP regimen may be an effective treatment for unresectable recurrent HCC.

In this study, the median PFS time was approximately 9 months, which was shorted than that of the previous reports. This may be caused by our inclusion population focusing on the patients who had an unresectable disease and higher tumor burden. Additionally, the ORR of the 16 patients who received the LEN-TAP regimen was 43.8%, which is lower than our data in advanced-stage primary HCC (data not given), and the reason behind this difference needs further exploration. Interestingly, we found out that combination therapy may be more effective in diffuse recurrent hepatocellular carcinoma, which was similar to the results of a recent publication (17). According to our data, more than half of the patients (10/16) had multiple intrahepatic lesions. After 4 months of treatment, the patients with multiple intrahepatic lesions had better ORR than those with single intrahepatic lesions according to mRECIST evaluation, even though the difference was not significant because of our small sample size. Therefore, we speculate that the LEN-TAP regimen may be the optimal solution for diffuse recurrent HCC, while the single-lesion urHCC may need extra local regional approaches such as radiotherapy, radiofrequency ablation, or microwave ablation.

According to our data, only one patient achieved conversion resection after 4 months of LEN-TAP treatment, which was significantly lower than the data in primary advanced-stage HCC according to previous reports (18). This may be partially because of the biological features of recurrent hepatocellular carcinoma and its special tumor microenvironment (16).

In this study, we also described the disease progression pattern and post-progression treatment schedule of these included patients. Although statistical inferences cannot be made due to the small sample size, the progression pattern is definitely related to the outcome of the patient (19–21). We need to enroll a large sample size cohort to further confirm this hypothesis. Furthermore, the best course of treatment after disease progression still needs further exploration.

According to our data, the most common adverse events in the 16 patients who accepted the LEN-TAP regimen were hypertension, abdominal pain, thrombocytopenia, diarrhea, and fatigue, which could be easily controlled. No grade 4 AE occurred in these patients, which may be explained by the dosage reduction of lenvatinib once grade 3 AE occurs. These data are similar to those of the previous studies in primary HCC (11, 18), and we concluded that the LEN-TAP regimen is safe and tolerable in urHCC patients.

Our investigation had some limits. First, this study was retrospective and single arm, and the sample size was relatively small. Therefore, it was difficult to make strong conclusions about these results. Second, our study was conducted in a single center and was a single-arm cohort lacking suitable controls. Finally, the median overall survival was not reached due to the short period of follow-up time. Consequently, it is necessary to conduct a prospective multicenter study in the future to validate our findings.

## Conclusions

The present study preliminarily confirmed that the LEN-TAP regimen consisting of TACE, lenvatinib, and camrelizumab was an effective and safe strategy in unresectable recurrent HCC. Further prospective studies are warranted to confirm these results.

## Data availability statement

The raw data supporting the conclusions of this article will be made available by the authors, without undue reservation.

## Ethics statement

The studies involving human participants were reviewed and approved by Ethics Committee of West China Hospital, Sichuan University. The patients/participants provided their written informed consent to participate in this study. Written informed consent was obtained from the individual(s) for the publication of any potentially identifiable images or data included in this article.

## Author contributions

CM and JS: study design, data collection, and manuscript writing and contributed equally to this work. XRZ: data collection. WP and XYZ: data collection and follow-up. TW study design and manuscript writing. All authors contributed to the article and approved the submitted version.
